# Functional Analysis of Continuous, High-Resolution Measures in Aging Research: A Demonstration Using Cerebral Oxygenation Data From the Irish Longitudinal Study on Aging

**DOI:** 10.3389/fnhum.2020.00261

**Published:** 2020-07-03

**Authors:** John D. O’Connor, Matthew D. L. O’Connell, Roman Romero-Ortuno, Belinda Hernández, Louise Newman, Richard B. Reilly, Rose Anne Kenny, Silvin P. Knight

**Affiliations:** ^1^The Irish Longitudinal Study on Aging, Trinity College, The University of Dublin, Dublin, Ireland; ^2^Department of Population Health Sciences, King’s College London, London, United Kingdom; ^3^The Global Brain Health Institute, Trinity College, The University of Dublin, Dublin, Ireland; ^4^Trinity Centre for Biomedical Engineering, Trinity College, The University of Dublin, Dublin, Ireland

**Keywords:** data-driven analysis, functional principal component analysis, near infrared spectroscopy, cerebral oxygenation, ageing, orthostatic hypotension

## Abstract

**Background**: A shift towards the dynamic measurement of physiologic resilience and improved technology incorporated into experimental paradigms in aging research is producing high-resolution data. Identifying the most appropriate analysis method for this type of data is a challenge. In this work, the functional principal component analysis (fPCA) was employed to demonstrate a data-driven approach to the analysis of high-resolution data in aging research.

**Methods**: Cerebral oxygenation during standing was measured in a large cohort [The Irish Longitudinal Study on Aging (TILDA)]. FPCA was performed on tissue saturation index (TSI) data. A regression analysis was then conducted with the functional principal component (fPC) scores as the explanatory variables and transition time as the response.

**Results**: The mean ± SD age of the analysis sample was 64 ± 8 years. Females made up 54% of the sample and overall, 43% had tertiary education. The first PC explained 96% of the variance in cerebral oxygenation upon standing and was related to a baseline shift. Subsequent components described the recovery to before-stand levels (fPC2), drop magnitude and initial recovery (fPC3 and fPC4) as well as a temporal shift in the location of the minimum TSI value (fPC5). Transition time was associated with components describing the magnitude and timing of the nadir.

**Conclusions**: Application of fPCA showed utility in reducing a large amount of data to a small number of parameters which summarize the inter-participant variation in TSI upon standing. A demonstration of principal component regression was provided to allow for continued use and development of data-driven approaches to high-resolution data analysis in aging research.

## Introduction

Measures of physiologic resilience are of increasing interest to the field of aging as they can identify an older person’s vulnerability to negative outcomes when faced with a stressor (Hadley et al., 2017; Varadhan et al., 2018; Kuchel, 2018). Assessment of resilience requires the tracking of a response to a stressor such as monitoring of the recovery of the cardiovascular system to standing from a supine position (Kim et al., 2011; Romero-Ortuno et al., 2011; McCrory et al., 2016; O’Connell et al., 2018). Improved technology in aging research can record stressor response at high spatial and temporal resolution. While this provides an opportunity to investigate physiological phenomena occurring in shorter timeframes and at smaller scales, it also presents a technical challenge for researchers in identifying the most appropriate methods to analyze the data. Recent publications are showing a growing interest in applying robust analysis methods for collinear, high-resolution data (Odden and Melzer, 2019; Wallace et al., 2019). An example of this type of data is generated in the study of orthostatic hypotension (OH) through monitoring of the neurovascular reaction to standing. Upon standing, both blood pressure and cerebral oxygenation show a rapid decline within 10–20 s after initiation (Finucane et al., 2017; O’Connor et al., 2020). This is followed by rapid recovery, with both signals returning to near baseline by 40 s after standing on average (Finucane et al., 2017; O’Connor et al., 2020). This is generally followed by a slower recovery over the next few minutes (Finucane et al., 2017; O’Connor et al., 2020).

An important risk factor for falls and other health outcomes, OH is assessed by measuring blood pressure recovery after standing (active stand protocol) or passive tilting (head-up tilt test; Kenny et al., 1986; Romero-Ortuno et al., 2013). The consensus definition for OH is a drop in systolic blood pressure (SBP) of ≥20 mmHg and/or a drop of ≥10 mmHg in diastolic blood pressure (DBP) within 3 min of standing (Kaufmann, 1996; Freeman et al., 2011). Traditionally, this was measured at discrete time points using a sphygmomanometer and more recently acquired continuously at high temporal resolution *via* beat-to-beat monitoring. The continuous monitoring of BP upon standing allows for an individual’s response profile to be examined in much greater detail. For example, assessment of the initial reaction to standing can reveal impairment; when SBP drops by ≥40 mmHg in the first 15 s this is referred to as initial orthostatic hypotension (Finucane et al., 2014, 2017; McCrory et al., 2016; O’Connell et al., 2018).

The effect of insufficiency in peripheral blood pressure may not translate to cerebral hypoperfusion due to the brain’s intrinsic mechanisms for maintaining sufficient cerebral tissue perfusion (i.e., cerebral autoregulation; CAR; Xing et al., 2017). Assessment of dynamic CAR, occurring in response to rapid changes in BP, has been described using Transcranial Doppler Ultrasound (TCD) to quantify cerebral blood flow (CBF) with quick deflation of a leg cuff driving changes in BP (Aaslid et al., 1989). A sit-to-stand maneuver can also be performed to invoke changes in systemic BP which allow the tracking of CAR (van Beek et al., 2008). Whereas TCD measures the CBF in the basal arteries of the brain, near-infrared spectroscopy (NIRS) may be used to measure cerebral oxygenation levels (Colier et al., 1997; Mehagnoul-Schipper et al., 2000; Kawaguchi et al., 2001; Kim et al., 2018; Mol et al., 2019). Although sit-to-stand or supine-to-stand tests are suitable for older adults, the speed of postural transition is heterogeneous and the hemeodynamic response is influenced by standing speed (de Bruïne et al., 2017; Mol et al., 2019; O’Connor et al., 2020). This is not unexpected given that much of the physiological response to standing is driven by muscle activation in the lower limbs (van Wijnen et al., 2017). In addition to the magnitude of central and peripheral hemeodynamic changes following orthostasis, the timing of nadir is of interest. Particularly when the speed of transition has been shown to vary from 2 to 27 s in older adults (O’Connor et al., 2020). Evaluation of CAR with aging may be confounded by the time taken to complete the postural transition. Commercial NIRS devices acquire data at a high temporal resolution, methodologies for analyzing these data have, however, focused on discrete time-points and feature identification with little consideration of timing differences (Briggs et al., 2018, 2019).

Functional principal component analysis (fPCA) is an established method for time-series analysis and data dimensionality reduction which can be used to identify complex trends in data including temporal changes. This work aimed to describe an application of fPCA to assess the association between transition speed during a supine-to-stand challenge and cerebral oxygenation in a large sample of older adults.

## Methods

### Cohort

The Irish Longitudinal Study on Aging (TILDA) began data collection in 2009, following a cohort (*n* = 8,504) of adults aged 50 and over as well as a small number of partners under the age of 50. Data is collected on health, social and financial circumstances (Donoghue et al., 2018). Trinity College Dublin granted ethical approval for the study, the design was compliant with the principles set out in the Declaration of Helsinki and participants provided informed, written consent. This work focuses on data collected from participants who completed TILDA’s health assessment center during the third wave of data collection (2014–2015). Since the primary aim of the article was to describe the implementation and interpretation of FPCA, all those with sufficient data were included in the analysis with a preference for adjusting for covariates as opposed to exclusion. Sample characteristics which have previously been associated with cerebral oxygenation such as depression and blood pressure are presented in Table 1 (Lucas et al., 2010; Briggs et al., 2019).

**Table 1:** Characteristics of the sample.

Age (years)	64.2 ± 7.9 [39–93]			-
Sex	Male: 45.8% (1,261)	Female: 54.3% (1,495)		-
Highest education achieved	Primary/none: 16.2% (445)	Secondary: 40.4% (1,113)	Third/higher: 43.5% (1,198)	-
Hypertensive	Yes: 35.3% (971)	No: 64.7% (1,781)		Missing: 4
Taking any anti-hypertensives (ATC C02, C03, C07, C08, C09)	Yes: 36.8% (1,015)	No: 63.2% (1,741)		-
CES-D (Centre for Epidemiological Studies Depression scale)	2.9 ± 3.5			Missing: 10
Taking at least one anti-depressant (ATC N06A)	Yes: 7.4% (203)	No: 92.6% (2,553)		-
Orthostatic hypotension	Yes: 13.1% (361)	No: 86.9% (2,395)		-
Smoking history	Current: 9.7% (266)	Past: 42.5% (1,172)	Never: 47.8% (1,318)	-
Body mass index	28.3 ± 4.7			Missing: 3

*Orthostatic hypotension was defined as those who had a blood pressure deficit of ≥20 mmhg systolic or ≥10 mmhg diastolic at 40 s after stand given that a previous study shows that most people have recovered by then (Finucane et al., 2017)*.

### Active Stand Protocol

Cerebral oxygenation measurements in TILDA were acquired in a quiet room with a controlled temperature of between 21°C and 23°C. During the active stand, a NIRS device (Portalite; Artinis Medical Systems, Zetten, Netherlands) was fixed to the forehead (3 cm lateral and 3.5 cm superior to the nasion) in approximately in the FP1 position of the ten-twenty electrode system (Klem et al., 1999). The Oxysoft (V3.0.53, Artemis Medical Systems, Zetten, Netherlands) software facilitated data collection and allowed manual signal marking at the beginning of a rest period. A digital photoplethysmography device (Finometer MIDI device, Finapres Medical Systems BV, Amsterdam, The Netherlands) was attached to each participant and used for measuring continuous blood pressure and identifying the timing of postural transitions *via* a built-in height correction unit. Data were acquired continuously while participants lay supine for 10 min before transitioning to a standing position and remaining standing for a further 3 min. The sampling frequency of the NIRS device was 50 Hz.

### Signal Processing and Analysis

Data from the NIRS files were extracted and processed using MATLAB (R2018a, TheMathWorks, Inc., MA, USA). The device recorded both HbO_2_ (μM) and HHb (μM), from which the summary measure of tissue saturation index (TSI) was calculated using Equation 1.

(1) $$TSI=\frac{HbO_{2}\times 100}{HbO_{2}+HHb}$$

Participants were removed from the analysis for missing/erroneous height sensor data or rest markers, as the time of their stand could not be reliably identified. After visual inspection of the signals, exclusions were applied for data suspected to be a measurement error—TSI falling below 10%, absolute HbO_2_ or HHb of less than 0.1 μM in over a quarter of data points, TSI change of over 45% overall, TSI change of less than 0.1% over first 30 s of stand and participants with a variation of more than 10% in the TSI baseline. Based on the manufacturer’s manual, those with an average fit factor (a measure of agreement between sensors on the NIRS device) of less than 98% were also removed. The time when the stand occurred was identified from the height sensor data, and the NIRS response profiles were subsequently aligned such that the zero second time-point corresponded to the point where the participant had started standing. Data was retained from 1 min before stand to 3 min after meaning each participant’s signal had 12,001 samples (i.e., 4 min of 50 Hz data).

### Functional Principal Component Analysis

#### Overview and Motivation

The fPCA was performed using a freely available MATLAB toolbox (Ramsay and Silverman, 2005; Ramsay et al., 2009). The purpose of applying fPCA, in this context, was to describe each participant’s response using a series of independent functions (i.e., functional principal components). These are defined by a functional loading across time for the NIRS recording which describes the influence of the signal at that time on the component. The functional principal component (fPC) score can then be calculated and is an estimation of the contribution of a component to an individual’s response. These scores may be standardized to Z-scores for interpretability where, for instance, a participant may have a score of +2SD on a specific fPC meaning their response was +2SD above the mean response for that component. This can then be interpreted graphically by plotting the mean ± 2SD of each of the components and is referred to in previous work as single component reconstruction (Brandon et al., 2013). To aid interpretation of the principal components, raw data were averaged over 10 s periods and a correlation matrix with the scores was produced. This enables the reader to assess which regions of the response are correlated with the components. Along with the graphical interpretation, scores can also be leveraged for statistical inference by using them as independent variables in regression (i.e., principal component regression).

#### Basis Functions, Knots and Smoothing

The first step of performing fPCA is fitting functions to the data. These functions are commonly defined by a linear combination of basis functions (e.g., Fourier, B-spline or wavelet bases). The choice of basis is dependent on the data input, Fourier bases are often used for periodic variation (e.g., seasonal weather changes or biomechanical phenomena repeating over a gait cycle) whereas B-splines may be used to model non-periodic processes such as the active stand (Ramsay and Silverman, 2005; Donoghue et al., 2008). The location of knots for B-splines is user-defined, they can be placed uniformly over the measuring window or located more densely at areas of the signal expected to show more variation (e.g., shortly after/before the stand in the data here). The smoothness of the function is often a subjective choice based on visual inspection of the fit of the function to individual participant data. Automated methods for choosing a smoothing parameter, such as cross-validation, exist but are recommended only for initial guidance (Ramsay and Silverman, 2002).

**Figure 1:**
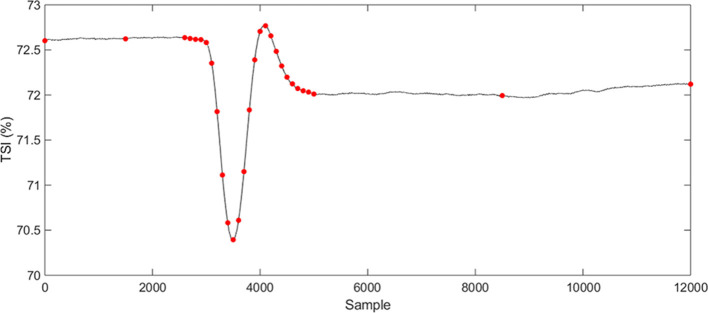
Placement of knots located preferentially around the area of the signal expected to show variation based on the mean response and previous works.

The NIRS data from the active stand is non-periodic and therefore B-splines were chosen as a suitable set of basis functions to describe the data. Knots were placed at 2-s intervals from 10 s before the stand to 40 s after with additional knots located at the beginning/end, 30 s before stand and 110 s after (Figure 1). This was based on the shape of the mean response and previous literature on cardiovascular responses to standing (Finucane et al., 2014; O’Connell et al., 2018). A smoothing parameter of 1e-8 was chosen based on the appropriate fit to the data and generalized cross-validation results (Ramsay and Silverman, 2005).

#### Functional Principal Components Regression

Regression analyses may be performed using the scores calculated from fPCA. It is important to decipher how many principal components to retain for analyses of this type. Common methods for achieving this are the scree plot (a graphical representation of the cumulative variance explained by fPCs), choosing components describing a defined proportion of the variance (e.g., 90%) or generalized cross-validation. Transition time (i.e., the amount of time taken to assume a standing position from supine) was used as an outcome variable in this demonstration and linear regression was performed with five fPCs as the explanatory variables. Covariates included in the model were: age, sex, use of antihypertensive or antidepressant medication, BMI and resting mean arterial pressure (MAP). The number of fPCs to use was chosen here by identification of the “elbow” in the scree plot.

In addition to analysis of data from TILDA, a demonstration is provided in Appendix 1 using simulated data to allow for easier implementation by other researchers (i.e., code may be used by replacing simulated data with experimental data). A MATLAB script for generating and analyzing the data is provided. The example of stand data includes a simulated trough magnitude as well as hypothetical timing of minimum, start and end of the nadir. A scree plot and single component reconstructions are presented for the simulated data (Appendix 1, Figures 5 and 6). A principal component regression (using the fPC scores in linear regression) is also performed for demonstration purposes on the simulated data with a theoretical dependent variable that is related to the simulated trough features.

**Figure 2:**
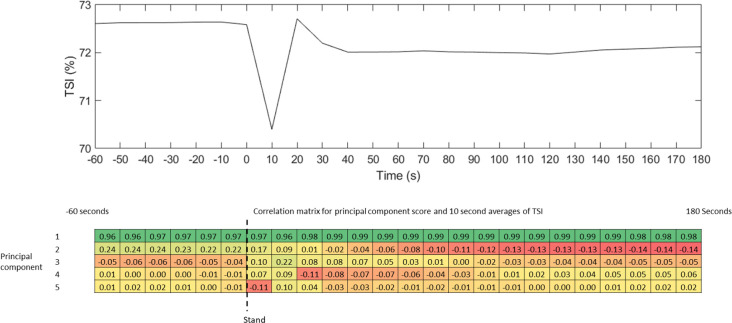
Correlation between 10-s averages of original tissue saturation index (TSI) data and principal component scores.

**Figure 3:**
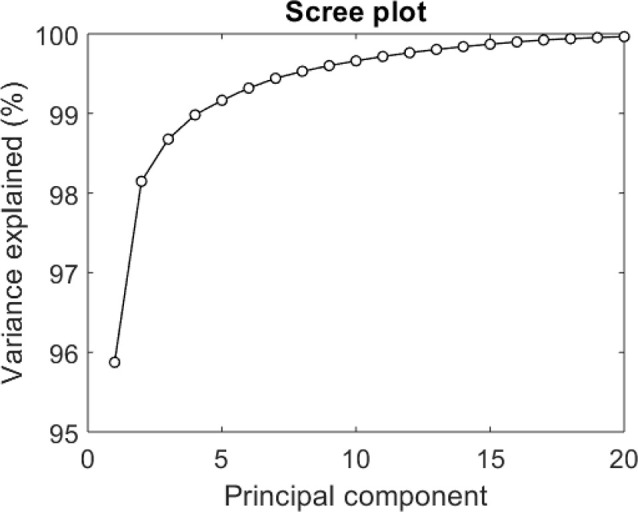
Scree plot for assessing cumulative variance explained by additional functional principal components (fPCs).

**Figure 4:**
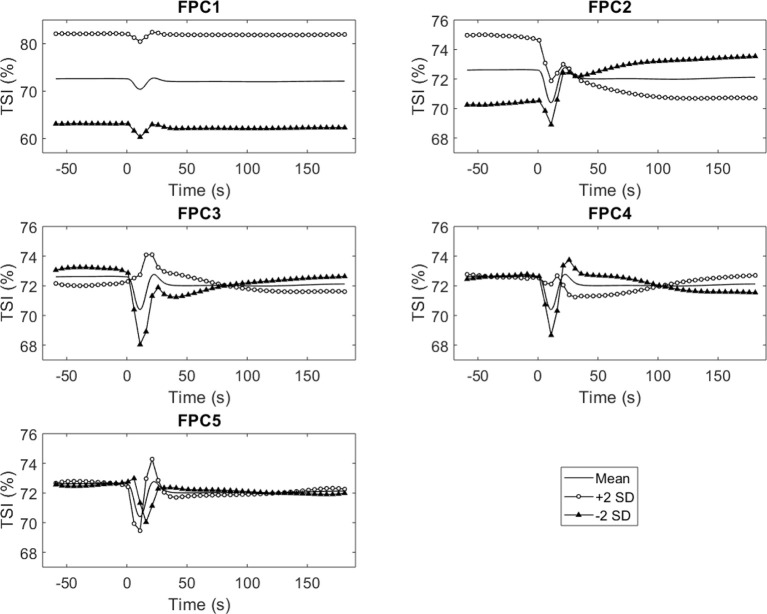
Graphical representations of the first five principal components for TSI (±2 standard deviations).

## Results

A total of 2,756 participants’ NIRS data were available for analysis (further detail provided in Appendix 2, Figure 7). Sample characteristics are detailed in Table 1. The mean (SD) age of the analysis sample was 64 (8) years. Females made up 54% and overall 43% had tertiary education. Levels of hypertension and antihypertensive use were similar at 35% and 37% respectively. The sample had a mean (SD) Centre for Epidemiological Studies Depression scale score of 2.9 (3.5) with 7% taking at least one antidepressant medication. Past smokers constituted 43% of the sample compared to 10% current and 48% never smokers. Body mass index was, on average, in the overweight range at 28 (5) kg/m^2^.

The mean TSI response for the full sample according to the 10 s averaged data consists of a sharp drop on standing followed by an initial recovery and stabilization throughout the rest of the stand (Figure 2). The correlation between the original data and the fPC scores is also shown in Figure 2 (this is analogous to loadings). Similar to previous studies, the “elbow” of the scree plot (Figure 3) was visually identified and 5 modes were chosen for retention (Woods and Edwards, 2007; Wilcox, 2012). The first principal component explained the majority of the variation in the TSI signal throughout the 4 min of rest and standing, accounting for 95.9% of the variation (Figure 3). As expected, the variance explained increased with the inclusion of more principal components with five modes explaining 99.2%.

The first component describes a baseline difference which shifts the entire trace up or down, this is evident in the component reconstruction for fPC1 (Figure 4) and the high correlations with all 10-s averages (Figure 2). The second principal component may be interpreted to represent the offset between baseline and recovery, i.e., how far above or below baseline the recovery stabilizes (Figure 4). This interpretation is supported by the changing sign in the correlation matrix during the transition from drop to recovery (Figure 2) as well as the difference between the baseline and final value on the single component reconstruction for fPC2 (Figure 4). Graphically, the third and fourth mode described the depth of the nadir/peak and the initial recovery (Figures 2, 4). Scores for the third, fourth and fifth components for TSI were linked to the difference between the nadir depth at around 10 s and initial overshoot at around 20 s as well as the temporal shift in where the minimum TSI occurred (Figures 2, 4). In particular, a low score for principal component five appeared to indicate a shifted minimum TSI value i.e., minimum TSI occurred after a longer duration following the transition to stand (Figures 2, 4). Linear regression analysis of the fPCs with the response variable of transition time showed associations between all but the first component (Table 2). The largest regression coefficients were for fPC4 and fPC5, suggesting that transition time is linked to the timing and depth of the nadir. When covariates were added to the model, the adjusted R^2^ value increased from 0.05 to 0.12 suggesting some of the variances in standing speed is driven by participant factors (Table 3). However, there remained significant relationships between the FPC4 and FPC5 and standing speed after adjusting for these factors (Table 3). Standing speed was associated with age, sex, BMI and antidepressant medication (Table 3).

**Table 2:** Results from linear regression with transition time as the dependent variable and functional principal components as the explanatory variables.

Transition time(s)	Coefficient	*t*-statistic	*P*-value	95% Confidence interval	
fPC1	−0.10	−1.90	0.058	−0.206	0.003
fPC2	0.11	1.98	**0.048**	0.001	0.210
fPC3	0.11	1.99	**0.047**	0.001	0.211
fPC4	0.28	5.30	**<0.001**	0.179	0.388
fPC5	−0.54	−10.13	**<0.001**	−0.647	−0.437
Intercept	7.26	135.91	**<0.001**	7.164	7.37

*Adjusted R^2^ = 0.05, *p*-values < 0.05*.

**Table 3:** Results from linear regression with principal component scores and covariates of age, sex, body mass index (BMI), antidepressants, antihypertensives and mean arterial pressure (MAP).

Transition time (s)	Coefficient	*t*-statistic	*P*-value	95% Confidence interval	
fPC1	−0.07	−1.36	0.173	−0.173	0.031
fPC2	0.04	0.81	0.420	−0.061	0.146
fPC3	0.08	1.44	0.151	−0.029	0.186
fPC4	0.17	3.16	**0.002**	0.065	0.276
fPC5	−0.47	−8.87	**<0.001**	−0.570	−0.363
Age	0.06	8.43	**<0.001**	0.045	0.072
Sex	0.63	5.37	**<0.001**	0.403	0.866
BMI	0.12	10.17	**<0.001**	0.095	0.140
Antidepressants	0.48	2.42	**0.016**	0.092	0.877
Antihypertensives	0.20	1.71	0.088	−0.029	0.429
MAP	−0.01	−1.46	0.144	−0.015	0.002
Intercept	−0.26	−0.35	0.723	−1.680	1.166

*Adjusted R^2^ = 0.12, *p*-values < 0.05*.

**Figure 5:**
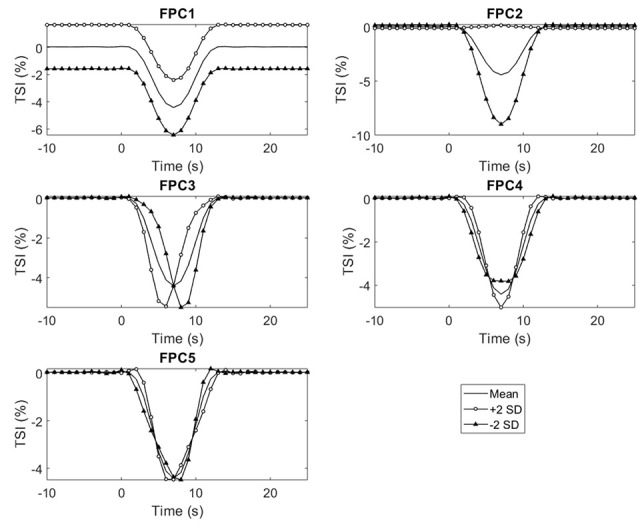
Single component reconstructions for the first five functional principal modes from simulated data.

**Figure 6:**
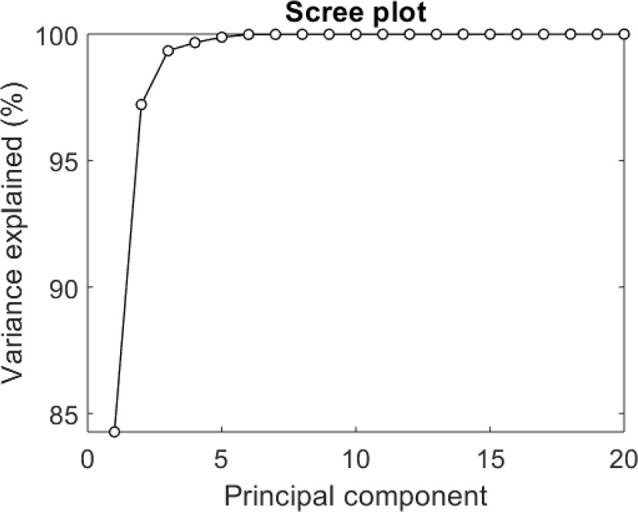
Scree plot showing the cumulative variance explained by each additional principal component in the simulated data.

**Figure 7:**
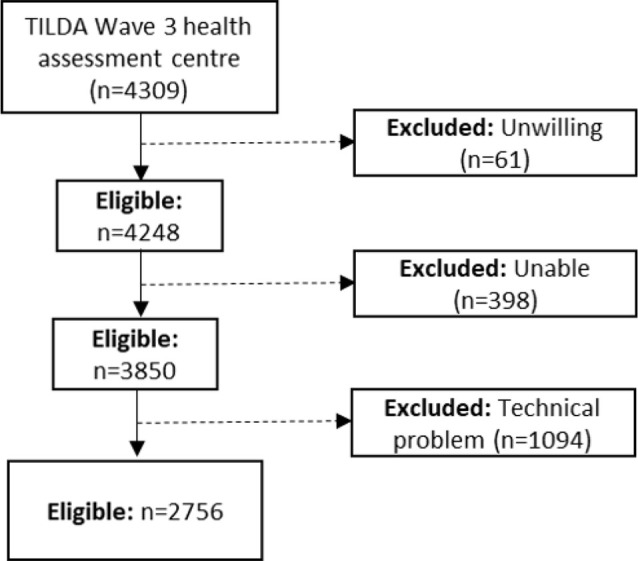
Exclusions for cerebral oxygenation data.

The first three components, resulting from fPCA on the simulated data (Figure 5 in Appendix 1), characterized the simulated features of baseline, trough depth and timing. The scree plot also shows that three components are the appropriate choice of fPCs to retain using the “elbow” identification method. Regression analysis showed significant relationships between the scores and simulated features.

## Discussion

The purpose of this work was to demonstrate a data-driven approach to the analysis of high resolution and collinear data as generated from dynamic physiological tasks in older adults. This was achieved using cerebral oxygenation data from an active stand protocol and fPCA.

The number of parameters needed to characterize an individual’s TSI response profile was reduced from several thousand time-points to five fPC scores which explained a large proportion of variance in the sample. The study demonstrates how component scores can be interpreted for use in further statistical analysis. In particular, the main interests of the active stand protocol were well characterized by the fPCs. Other aspects of the response such as the depth of the initial drop or the timing of the peak drop were also well described by subsequent components. Transition time demonstrated an association with several fPC scores related to the timing and magnitude of the nadir after standing. This is notable as identification of timing differences may be difficult to detect with traditional analysis methods.

The study of the orthostatic response to standing from supine is of interest in older adults given its relationship with falls, depression and mortality (McCrory et al., 2016; Finucane et al., 2017; Briggs et al., 2019). Given the range in older adult mobility, the speed at which the transition is completed is an important source of variation that is associated with both timing and magnitude of hemeodynamic parameters (de Bruïne et al., 2017; Mol et al., 2019; O’Connor et al., 2020). The musculoskeletal effort of standing causes vasodilation in the lower limbs (as evidenced by larger changes during active vs. passive stand; van Wijnen et al., 2017). A faster transition may lead to quicker dilation of the vasculature and has shown larger changes from baseline in TSI and blood pressure (O’Connor et al., 2020). A slower transition can lead to a delayed nadir in orthostatic measures and the identification of temporal differences (FPC4 and FPC5) in this work are important for the interpretation of previous studies which assume minimum values occur at the same time after stand (e.g., 10 s after stand). The time taken to transition is related to aging, sex, BMI, and antidepressant medication use; therefore, adjustments should be made for analysis of cerebrovascular function in aging using supine-to-stand tests.

The data sampling rate has been increasing in many areas of aging research including during continuous monitoring of blood pressure and cerebral oxygenation (Kawaguchi et al., 2001; Briggs et al., 2018, 2019; O’Connell et al., 2018). Commonly, data reduction is achieved *via* time point averaging (e.g., 10-s averages), however, given the relatively high sampling frequency capabilities of commercially available devices this leads to most of the collected data being discarded. As such, complex trends may be difficult, if not impossible to identify with this type of analysis; fPCA can reveal such trends in the data (e.g., temporal trends such as shifting peak/drop associated with fPC5). Principal component regression also addresses the issue of collinearity, with the ability to provide a small number of continuous independent parameters which characterize the entire response profile. The analysis pipeline demonstrated in this work may prove useful to researchers wishing to investigate links between high-resolution, collinear data collected in research into aging and outcome measures. This type of analysis, with additional development, may ultimately prove useful in a clinical setting for automated analysis of NIRS data, identifying subtle trends in the active stand response which may be associated with poor or desirable outcomes. Starting to address limitations in modeling methods for physiologic resilience tests can facilitate the development of improved prediction for important outcomes such as falls, cognition or mortality (Wallace et al., 2019).

A large proportion of the variance between individuals was explained by the first component (96%) suggesting that the differences in baseline dominated. This is similar to trends in other hemeodynamic measures during standing such as heart rate and blood pressure. However, the proportion of variance explained by the baseline shift is larger in the case of TSI given the stand elicits an attenuated reaction compared to that of BP (i.e., TSI changes of 1–2% whereas BP changes by around 25%; O’Connor et al., 2020). This smaller change of TSI is expected given the action of cerebral autoregulation and changes of this magnitude have shown an association with outcomes, such as depression, in TILDA (Briggs et al., 2019). The remaining components (FPC2–FPC5) cover a small proportion of the variance (3.2%) but represent the changes in cerebral oxygenation brought about by standing. These components were useful in predicting standing time in the study and previous works have shown that components with low variance explained can still be crucially important (Jolliffe, 1982).

Notwithstanding these advantages, the analysis of NIRS data in this manner does have some limitations. Firstly, the selection of the number of components to use to adequately represent the data can be challenging, although objective criteria do exist to assist in making this selection (Jolliffe, 1982). Additional methods such as cross-validation or parallel analysis may be required for robust identification of the number of components needed for identifying relationships with specific outcomes. Secondly, the direct effect of NIRS measurements at a specific time-point on the dependent variable may be difficult to decipher given that several components (which are influenced by the same time point) can be related to the outcome measure simultaneously. Clinicians may find it difficult to identify modes of variation in the data by eye; however, this can be facilitated by the development of a software package to fit the fPCA model described herein to a particular individual’s measured response profile. The principal components presented here are generated from those who attended TILDA’s health center and had valid data for NIRS. To ensure population representativeness, considering sample loss and non-attendance, weighting should be considered when the method is applied to specific outcome measures. Systems using NIRS can produce reliable estimates of CAR during orthostasis and have shown utility in clinical studies using head-up tilt testing (Bachus et al., 2018; Mol et al., 2020). There is a high correlation between CAR assessed using either TCD or NIRS (Zweifel et al., 2010). The NIRS system in this study has an estimated penetration depth of 20 mm into the prefrontal cortex, future work assessing the correlation between oxygenation in this area and other regions of the brain is of interest.

## Conclusion

In conclusion, fPC regression shows utility in identifying independent parameters which characterize the cerebral oxygenation reaction to standing. A demonstration of principal component regression was provided to allow for continued use and development of data-driven approaches to high-resolution data analysis in aging research.

## Data Availability Statement

The datasets for this study are available upon reasonable request to The Irish Longitudinal Study on Ageing (email: tilda@tcd.ie).

## Ethics Statement

The studies involving human participants were reviewed and approved by Trinity College Dublin. The patients/participants provided their written informed consent to participate in this study.

## Author Contributions

JO’C: drafting manuscript. JO’C, SK, and LN: data analysis. LN and RK: data acquisition. JO’C, MO’C, BH, LN, RR-O, RR, RK, and SK: study concept, interpretation of data, critical revision of the article for important intellectual content, and final approval for submission.

## Conflict of Interest

The authors declare that the research was conducted in the absence of any commercial or financial relationships that could be construed as a potential conflict of interest.

## Appendix

### Appendix 1

This section includes a Github link (https://github.com/JohnDOConnor/TILDA_bioengineering) to code for performing principal component regression. Simulated data is used here for demonstration. Each simulated signal has a random trough time, magnitude and baseline. The component reconstructions demonstrate the ability to identify these trends in the first 3 components, the scree point also shows little benefit from including more than 3 components.
